# Case report of a successful conservative surgical treatment of a giant liposarcoma of the forearm invading the median nerve

**DOI:** 10.1016/j.radcr.2023.12.002

**Published:** 2024-01-27

**Authors:** Jamal Karbal, Amine Machmachi, Walid Bouziane, Moncef Amahtil, Ousmane Laye Diene, Abdelkrim Daoudi

**Affiliations:** Traumatology-Orthopedics Department, Mohammed VI University Hospital Center, Faculty of Medicine and Pharmacy Mohammed First University Oujda, Oujda, Morocco

**Keywords:** Differentiated liposarcoma, Lipoma-like, Resection, Forearm

## Abstract

Liposarcomas (LPS) are the most common malignant tumors among soft tissue sarcomas. We report a rare case of well-differentiated liposarcoma (lipoma-like) located along the anterior compartment of the right forearm, which was successfully treated with complete excision, resulting in good functional recovery without recurrence during a 2-year follow-up. Surgery is the primary treatment for localized forms, involving complete and radical excision with clear resection margins. LPS represents a heterogeneous group of soft tissue sarcomas, with diagnosis primarily relying on histological examination. Their care requires a multidisciplinary approach.

## Introduction

Liposarcoma (LPS) of the extremities is the second most common soft tissue sarcoma after malignant fibrous histiocytoma, representing 16%-18% of all malignant soft tissue tumors [1], [2], [3], [4], [5]. MRI plays a significant role in diagnosis in the majority of cases due to the presence of fatty components. We present the results of a 48-year-old patient with a large liposarcoma in the anterior compartment of the forearm, in proximity to the median nerve. The patient underwent complete resection, resulting in good functional recovery with no recurrence.

## Case report

**Fig. 1:**
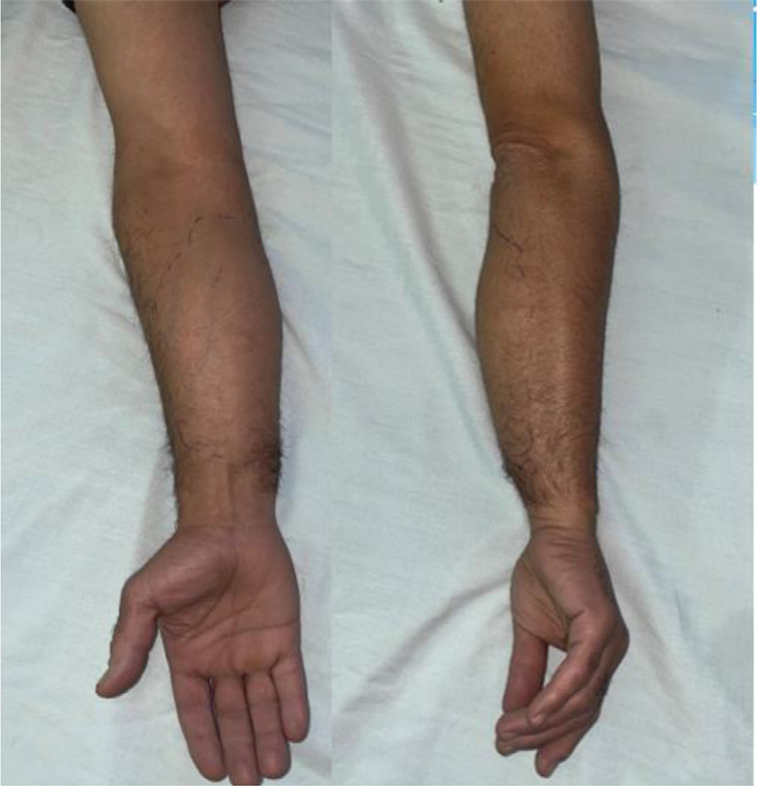
Giant anterior swelling of the right forearm.

**Fig. 2:**
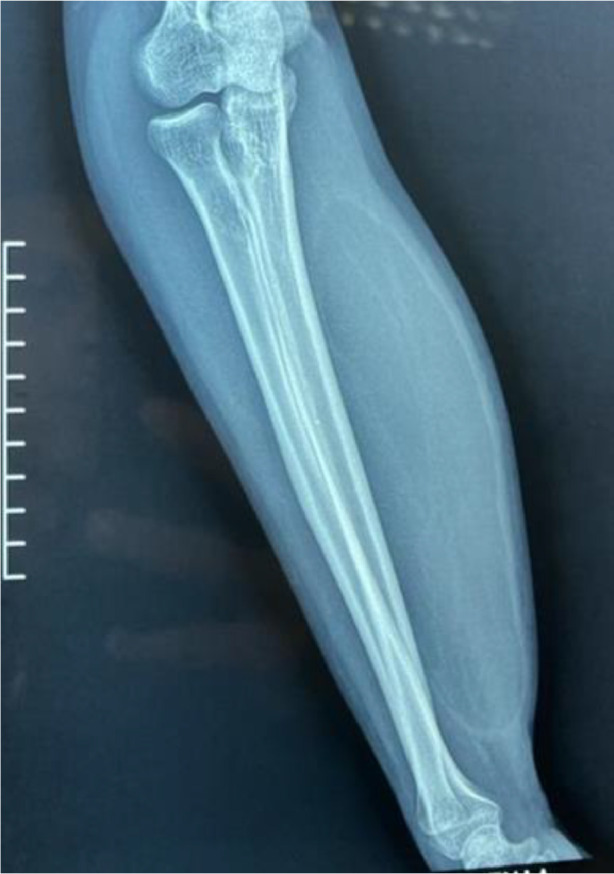
Standard X-ray of the forearm showing no anomalies.

**Fig. 3:**
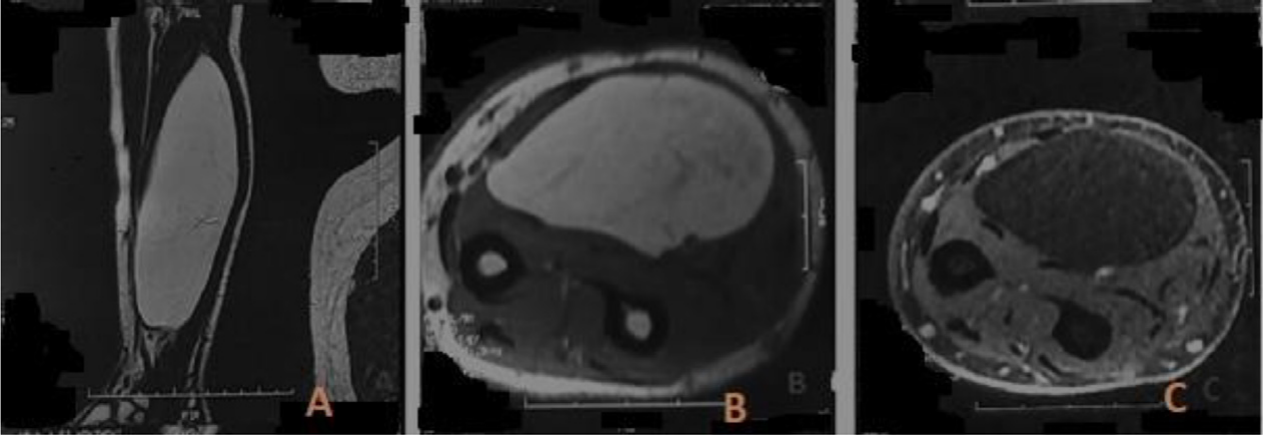
Well-defined homogeneous tumoral process in the anterior compartment of the forearm, subaponeurotic: (A) hyperintense, (B) fat tissue signal, (C) hypointense in contact with the median nerve.

We report the case of a 48-year-old patient with no significant medical history who presented with an enormous swelling on the right forearm (Fig. 1). This swelling had been present for 1 year and had progressively increased in size to become massive. It caused significant discomfort due to its weight and limited manual activities. The patient also experienced pain and paresthesia radiating into the thenar eminence. Upon clinical examination, the tumor was massive, occupying the entire anterolateral region of the forearm, and measuring 14 cm along its major axis. It was firm to the touch and movable in relation to the deep tissues. There was a decreased sensitivity on the palmar side of the index and thumb, but no motor deficits were observed. The initial radiological assessment, including standard X-rays of the forearm in both anteroposterior and lateral views, revealed no anomalies (Fig. 2). An ultrasound examination showed a well-defined fatty soft tissue mass measuring 86 × 46 mm. To further evaluate the condition, an MRI was performed, revealing a fatty, well-demarcated tumoral process within the anterior compartment of the forearm along the superficial flexors. This mass had close contact with the median nerve, raising suspicion of a lipoma or liposarcoma (Fig. 3).

**Fig. 4:**
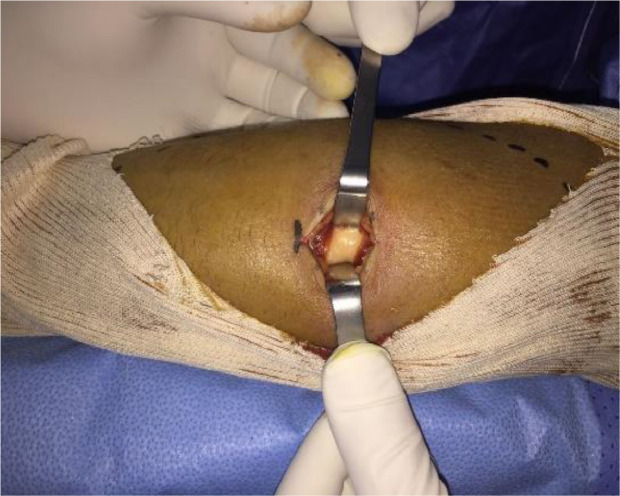
Intraoperative image of the biopsy.

**Fig. 5:**
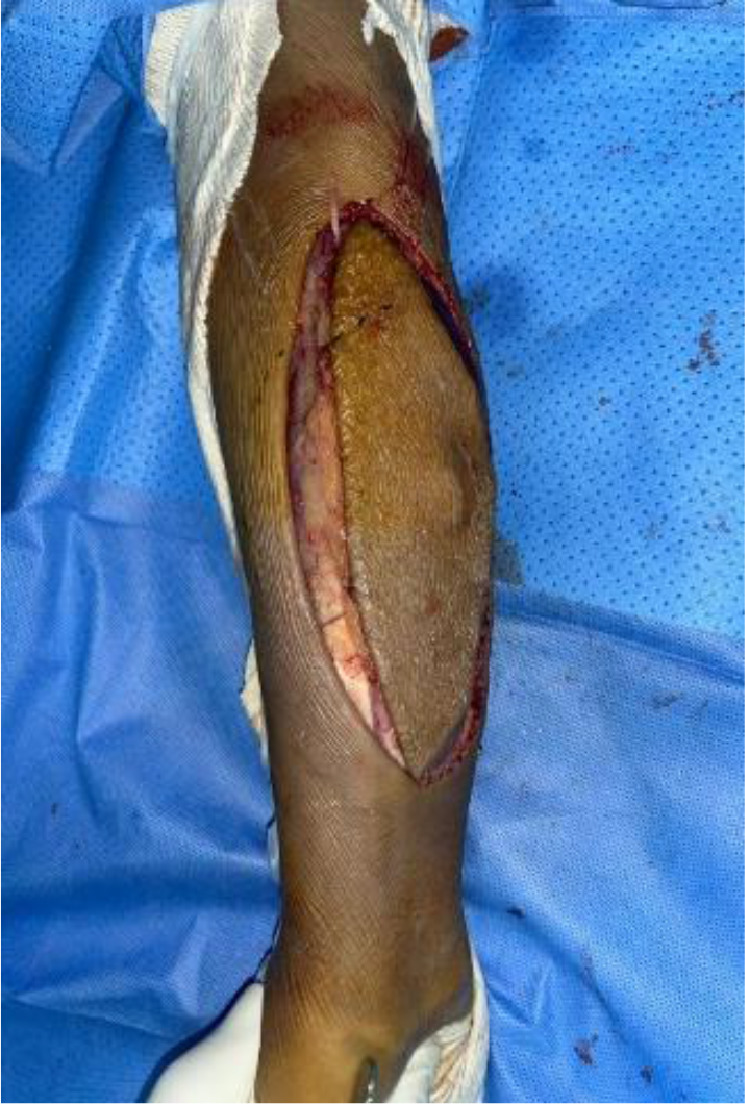
Total tumor resection.

**Fig. 6:**
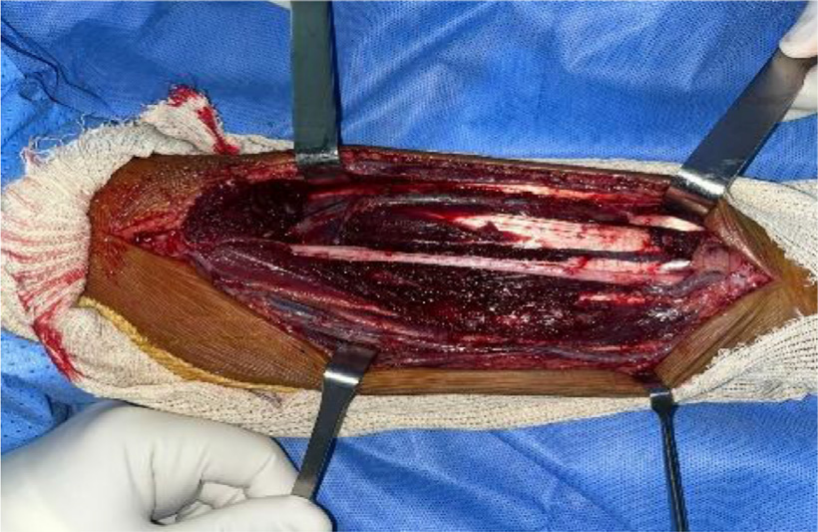
Intraoperative image after complete resection while preserving the median nerve.

**Fig. 7:**
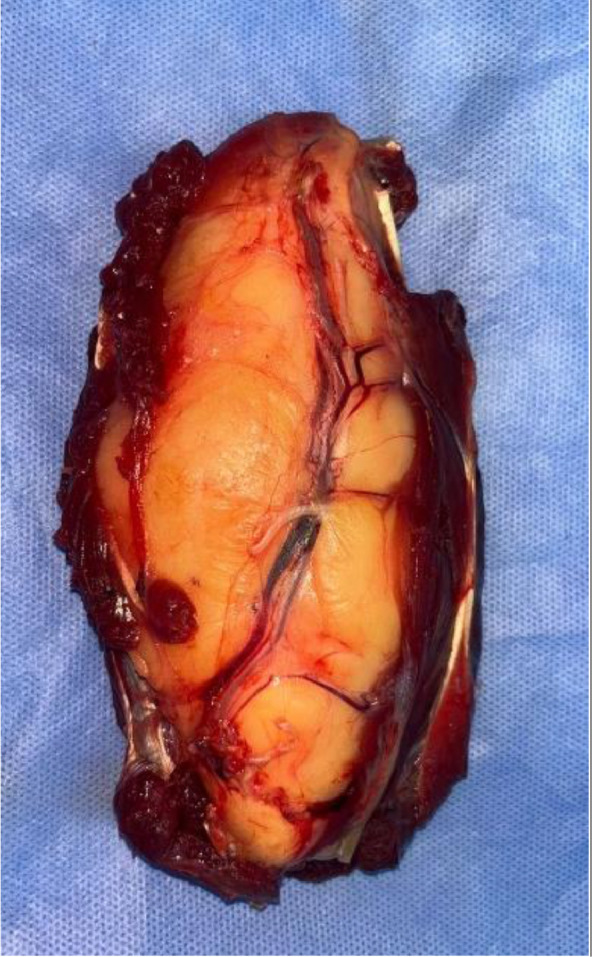
View of the tumor specimen.

**Fig. 8:**
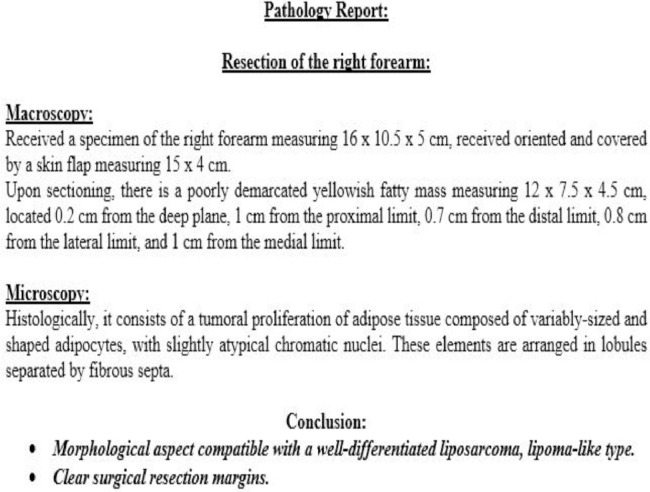
Histological confirmation results after tumor excision.

A biopsy was initially conducted, and the results were consistent with a well-differentiated liposarcoma (Fig. 4). Metastatic workup yielded negative results. Subsequently, the chosen therapeutic approach was a conservative surgical procedure, involving en bloc resection of the tumor with a 2 cm safety margin in width and depth. This procedure also required the complete resection of the superficial flexors of the anterior forearm compartment while preserving and meticulously dissecting the median nerve. A tenodesis of the remaining superficial flexors with the deep flexors was performed to maximize finger mobility (Fig. 5, Fig. 6, Fig. 7). The histological examination of the operative specimen confirmed the diagnosis of a well-differentiated liposarcoma of the lipoma-like subtype with clear surgical margins (Fig. 8).

**Fig. 9:**
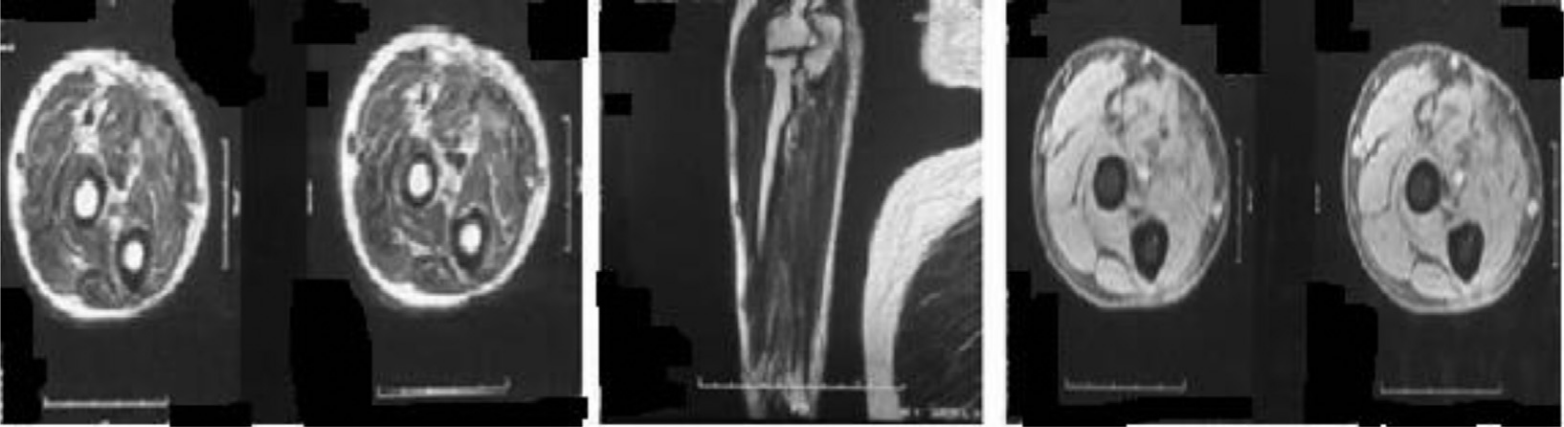
Follow-up MRI sequences showing no signs indicative of tumor recurrence.

The patient had undergone clinical and radiological follow-up with an MRI, which showed the absence of any signs of local recurrence after a 2-year follow-up (Fig. 9). Emphasis was placed on self-rehabilitation of the hand and fingers, leading to a good functional recovery and a return to work after three months.

## Discussion

Liposarcomas (LPS) are the most common malignant tumors among soft tissue sarcomas and account for approximately 10%-35% of those originating from primitive mesenchymal cells. They typically occur in adults between the ages of 40 and 60, with a higher incidence in males [6]. These tumors are primarily deep-seated, located within or between muscles [[7], [8], [9], [10], [11], [12]]. Clinically, like other soft tissue sarcomas, LPS often manifest as painless soft tissue masses. However, approximately 10%-15% of cases may present with pain [1], [2], [3]. In our case, the mass was located in the superficial finger flexors.

MRI plays a crucial role in investigating LPS, which typically presents as a hypointense signal in T1 and a homogeneous hyperintense signal in T2, with well-defined boundaries, as seen in our case. In addition to its value in assessing local and regional extension (involvement of bone, soft tissues, and vascular-nerve structures), a definitive diagnosis of these tumors can only be made through histological means, requiring a surgical biopsy to be performed before any therapeutic intervention [13].

According to the World Health Organization (WHO), LPS is histologically classified into five groups: well-differentiated, myxoid, pleomorphic, dedifferentiated, and mixed [[1], [2], [3], [4], [5], [6], [7], [8], [9], [10], [11], [12]]. Well-differentiated liposarcoma is the most common histological subtype, accounting for approximately 40%-50% of soft tissue LPS cases [[1], [12], [14], [15]]. Within this type of LPS, five subtypes are distinguished: [16]

• Well-differentiated adipocytic LPS, also known as lipoma-like, diagnosed by the presence of hyperchromatic atypical cells. This is the histological type of our case, confirmed by the tumor resection specimen.
• Well-differentiated sclerosing LPS characterized by the presence of fibrous septa that dissect mature adipose tissue, containing atypical cells with pleomorphic and hyperchromatic nuclei.
• The “inflammatory” variant, characterized by an intratumoral inflammatory infiltrate of polymorphonuclear or lymphoplasmacytic cells, which may raise differential diagnoses with inflammatory or lymphomatous conditions.
• Well-differentiated spindle cell LPS, a variant described, primarily affecting subcutaneous tissue, especially in the shoulders. It is composed of spindle cells with hyperchromatic, minimally atypical nuclei, often associated with irregular adipocytes and some lipoblasts. This subtype may pose a differential diagnosis challenge with spindle cell lipoma.
• Dedifferentiated LPS, which always arises from a well-differentiated LPS and is associated with a high-grade nonlipogenic sarcomatous component. This component can resemble malignant fibrous histiocytoma (MFH) with storiform pleomorphic features or fibrosarcoma. Diagnosing this type can be challenging when dealing with biopsies that only involve poorly differentiated areas. Adequate sampling to identify well-differentiated areas is crucial for making this diagnosis.

Surgery remains the primary treatment for localized LPS. Only complete and radical excision with clear resection margins offers a potential curative approach. Postoperative radiotherapy can reduce the risk of recurrence [17]. It is particularly indicated when incomplete resection leaves tumor cells behind to sterilize them. Additionally, it may be considered as palliative treatment for inoperable large tumors. Myxoid LPS is the most radiosensitive subtype. Chemotherapy has limited utility; however, it appears to improve survival rates and reduce the incidence of local recurrences and metastases in larger tumors. In our case, the treatment involved surgical resection of the tumor while preserving the median nerve. The surgery followed ten sessions of radiotherapy to shrink the mass before resection, although the radiotherapy was ineffective in this particular case.

The histological type and grade of the tumor are the primary prognostic factors, and they are correlated with the risk of local recurrences and metastases [18]. Other prognostic factors that have been implicated include the quality of excision, the presence of synchronous metastases, as well as neurovascular and bone involvement [19].

## Conclusion

LPS presents challenges in terms of diagnosis and therapeutic management, highlighting the importance of a multidisciplinary, early, and grade-specific decision that aligns with the stage of the condition. This approach enables the possibility of long-term remission.

## Patient consent

Written informed consent was obtained from the patient for publication of this case report and accompanying images. A copy of the written consent is available for review by the editor-in-chief of this journal on request.
